# Interleukin 37 Suppresses M1 Macrophage Polarization Through Inhibition of the Notch1 and Nuclear Factor Kappa B Pathways

**DOI:** 10.3389/fcell.2020.00056

**Published:** 2020-02-14

**Authors:** Peitao Zhou, Qianqin Li, Shuwen Su, Wenhui Dong, Suyu Zong, Qiong Ma, Xi Yang, Daming Zuo, Shaoyi Zheng, Xianzhong Meng, Dingli Xu, Qingchun Zeng

**Affiliations:** ^1^Key Laboratory for Organ Failure Research, Department of Cardiology, Nanfang Hospital, Southern Medical University, Guangzhou, China; ^2^Department of Cardiovascular Surgery, Nanfang Hospital, Southern Medical University, Guangzhou, China; ^3^Department of Immunology, School of Basic Medical Sciences, Southern Medical University, Guangzhou, China; ^4^Department of Surgery, University of Colorado Denver, Aurora, CO, United States; ^5^Guangzhou Regenerative Medicine and Health Guangdong Laboratory, Guangzhou, China

**Keywords:** interleukin 37, macrophage polarization, Notch1, NF-κB, calcific aortic valve disease

## Abstract

Macrophage-orchestrated chronic inflammation plays an important role in cardiovascular disease, including accelerating the development of calcific aortic valve disease (CAVD). M1 and M2 macrophage polarization imbalances can alter intensity of inflammatory responses. Recombinant human interleukin 37 (IL-37) could be involved in regulating immune cell function to attenuate inflammation. This study aimed to identify IL-37 specifically modulates M1 polarization and investigate the underlying mechanism. Compared with normal valves, there are more M1 macrophages accumulation and less IL-37 expression in calcific aortic valves, which may indicate a negative relationship between IL-37 and M1 polarization. THP-1 cells could differentiate into resting macrophages with phorbol-12-myristate-13-acetate (PMA) and then polarize into M1 macrophages following treatment with lipopolysaccharide (LPS) and interferon gamma (IFN-γ). *In vitro*, recombinant human IL-37 attenuated the expression of inducible nitric oxide synthase (iNOS), CD11c, IL-6 and monocyte chemoattractant protein 1 (MCP-1) in M1 but augmented the expression of CD206 and IL-10 in M2. The suppression of M1 polarization was associated with the inhibition of the activation of the nuclear factor kappa B (NF-κB) and Notch1 signaling pathways. These results demonstrated that IL-37 inhibits the macrophages polarizing into M1 type via the inhibition of the Notch1 and nuclear factor kappa B pathways. In summary, IL-37 could be a potential therapeutic candidate for progressive CAVD by modulating M1 polarization and its orchestrated inflammation.

## Introduction

Calcific aortic valve disease (CAVD) is recognized as a chronic progressive inflammatory disease and has become a leading cause of cardiovascular disease in people aged 65 years or older and the second most common indication for cardiac surgery (Mohler et al., 2001; Rajamannan et al., 2011). However, the mechanism underlying CAVD development remains incompletely understood. But examinations of calcific human aortic valves indicate that macrophages may play an important role in the calcification process (Hjortnaes et al., 2010).

Macrophages are myeloid immune cells that coordinate inflammatory processes. Macrophages could assume two major functional phenotypes: the classical activation phenotype, also called M1 macrophages, and the alternative activation phenotype, also known as M2 macrophages (Liu et al., 2014). M1 macrophages can be induced by Toll-like receptor (TLR) ligands and IFN-γ while M2 by IL4/IL13 (M2a), immune complex (M2b), and the anti-inflammatory cytokines IL-10 or transforming growth factor-β (M2c) (Zhou et al., 2014). M1 macrophages express numerous pro-inflammatory cytokines, such as IL-1β, IL-6, IL-23, inducible nitric oxide synthase (iNOS) and tumor necrosis factor alpha (TNF-α), while M2 macrophages secrete molecules including Arginase1 (Arg1), IL-10 and transforming growth factor-β1 (TGF-β1) which are associated with parasite infestation, tissue remodeling and tumor progression (Sica and Mantovani, 2012; Liu et al., 2014). Notably, studies have shown that M1 and M2 are found in the central portions of atherosclerotic and calcific lesions and convert into each other in response to micro-environmental changes (Mantovani et al., 2013; Liu et al., 2014). The finding that M1 macrophages accumulate in aortic valvular lesions suggests that the inflammation induced by M1 macrophages is pivotal in cardiovascular calcification (Li et al., 2016).

However, the mechanism underlying macrophage polarization is complicated and may involve various signaling pathways that affect one another. Many signaling pathways, such as Akt2 (Liu et al., 2019), activator protein 1 (AP-1) (Liu et al., 2014), interferon-regulatory factor (IRF) (Satoh et al., 2010), peroxisome proliferator-activated receptor gamma (PPAR-γ) (Odegaard et al., 2007), hypoxia-inducible factor -2alpha (HIF-2α) (Wang et al., 2018), nuclear factor kappa B (NF-κB) (Oeckinghaus et al., 2011) and Notch1 signaling pathways (Wang Y. et al., 2010), are involved in macrophage polarization. The stimulation by lipopolysaccharide (LPS) plus interferon gamma (IFN-γ) induces the polarization of resting macrophages into activated M1 macrophages (Foldi et al., 2016).

IL-37, also known as IL-1F7, a member of the IL-1 family, has emerged as a potential suppressor of inflammation (Dinarello and Bufler, 2013). The protein has been reported in human monocytes, macrophages, epithelial cells, aortic valve interstitial cells, tonsil plasma cells and breast carcinoma cells, but has not been detected in murine (Nold et al., 2010). IL-37 has been observed to exert anti-inflammatory effects on the innate and acquired immune responses both *in vitro* and *in vivo* (Boraschi et al., 2011). Recombinant human IL-37 attenuates pro-inflammatory cytokine production in mouse RAW cells, monocytes and epithelial cells and alleviates inflammation-induced injury in wild-type mice (Nold et al., 2010; Dinarello et al., 2016). Notably, our groups have proven that IL-37 transgenic mice are protected from the aortic valve lesions induced by inflammation (Zeng et al., 2017). However, whether IL-37 suppresses macrophage polarization to inhibit inflammation has not yet been clearly determined.

In this study, we aimed to determine whether IL-37 suppresses M1 polarization to inhibit inflammation and to explore the mechanism by which IL-37 exerts its effect. We examined the expression of the M1/M2 macrophage phenotypes in calcific and non-calcified aortic valves, evaluated the effect of recombinant IL-37 on macrophage polarization and investigated whether IL-37 modulates M1 macrophage polarization via the NF-κB and Notch1 pathways.

## Materials and Methods

### Cell Culture and Treatment

THP-1 cells were obtained from the American Type Culture Collection (ATCC). Recombinant human IL-37 (Cat. No. Ab224789) was purchased from Abcam. THP-1 cells were maintained in Roswell Park Memorial Institute (RPMI) 1640 medium (Gibco) supplemented with 10% fetal bovine serum (FBS; Gibco) at 37°C in a humidified incubator with a 5% CO2 atmosphere. THP-1 cells were seeded at 5 × 10^6^ cells/well in 6-well plates and cultured in RPMI 1640 medium containing 10% FBS and then were differentiated into resting (M0) macrophages by 24 h incubation with 100 ng/ml phorbol 12-myristate 13-acetate (PMA; Sigma-Aldrich, Cat. No. 79346) followed by 24 h incubation in RPMI medium (Genin et al., 2015). After culturing for 48 h, cells were washed with phosphate-buffered saline (PBS), following by treating with or without LPS (100 ng/ml; Sigma-Aldrich, Cat. No. L2630) and IFN-γ (20 ng/ml; Sigma-Aldrich, Cat. No. SRP3058) for 24 h to polarize into M1 macrophages (Genin et al., 2015; Tedesco et al., 2015). To determine the effect of IL-37 on M1 polarization, we pre-treated the M0 macrophages with IL-37 (0.1 ng/ml) (Zeng et al., 2017) 1 h before adding LPS and IFN-γ to the medium (Zhou et al., 2015).

To determine the effects of NF-κB and Notch1 on M1 polarization, we added NF-κB specific inhibitor BAY11-7082 (5 μM; Sigma-Aldrich, Cat. No. B5556) and the γ-secretase inhibitor DAPT (50 μM; Sigma-Aldrich, Cat. No. D5942) to the culture medium 1 h before adding LPS and IFN-γ to the medium. To investigate the effect of Notch1 on NF-κB phosphorylation, we added DAPT (50 μM) to the culture medium 1 h prior to treating the cells with LPS and IFN-γ.

### Histology and Immunohistochemistry

This study was approved by the Ethical Committee of Nanfang Hospital, China. Informed consent was obtained from all patients. Normal aortic valves were collected from the explanted hearts of six males (mean age 58 ± 8.1 years) without CAVD undergoing heart transplantation. Valves with calcification were obtained from 6 males (mean age 60 ± 11.3 years) undergoing aortic valve replacement.

Paraffin-embedded non-calcific and calcific aortic valve samples were cut into 5-μm-thick sections, and then were incubated for 20 min at 65°C before deparaffinization with xylene and alcohol. Hematoxylin and eosin-stained sections were examined to identify the difference between non-calcified and calcified aortic valves. For Immunohistochemistry, following antigen retrival through microwave, the prepared sections were incubated in 3% H_2_O_2_ for 10 min. Then the sections were rinsed with phosphate buffer saline and blocked in 5% bovine serum albumin (BSA) for 30 min at room temperature followed by incubation with primary antibodies against CD11c (1:200; Abcam, Cat. No. EP1347Y), CD206 (1:1000; Abcam, Cat. No. ab8918) and IL-37 (1:100; Abcam, Cat. No. ab101376) overnight at 4°C and horseradish peroxidase-conjugated secondary antibody for 30 min at room temperature. And then diaminobenzidine (DAB) was used as a chromogen to visualize positive cells. For each valve, integrated optical density (IOD)/area was counted in five representative high-powered fields in each of three slides through Image-Pro Plus 7.0. For each field, areas of interest were selected to measure IOD and area. Moreover, background IOD/area value from the directly measured IOD/area value was subtracted to acquire more accurate IOD/area value. Then, the mean value of these 15 fields was used to evaluate the target protein expression.

### Immunoblotting

Briefly, total protein of aortic valve tissue samples and cell lines was extracted using protein extraction reagent (Thermo Scientific) with a cocktail of proteinase inhibitors (Fude Biological Technology) and a cocktail of phosphatase inhibitors (Fude Biological Technology) according to its protocol. The proteins were separated by 8–12% SDS-PAGE and then transferred onto PVDF membranes. After blocking with 5% non-fat dry milk solution or 5% bovine serum albumin solution for 1 h at room temperature, the membranes were incubated with antibodies against iNOS (1:1000; Cell Signaling Technology, Cat. No. 2977), CD11c (1:1000; Abcam, Cat. No. EP1347Y), CD206 (1:1000; Abcam, Cat. No. ab8918), IL-37 (1:1000; Abcam, Cat. No. ab153889), IL-6 (1:1000; Abcam, Cat. No. 12153), MCP-1 (1:1000; Cell Signaling Technology, Cat. No. 39091), NICD1 (1:1000; Cell Signaling Technology, Cat. No. 3608), phosphorylated (1:1000; Cell Signaling Technology, Cat. No. 3033) and total NF-κB (1:1000; Cell Signaling Technology, Cat. No. 8242) and GAPDH (1:1000; Cell Signaling Technology, Cat. No. 5174) overnight at 4°C followed by incubation with horseradish peroxidase-conjugated secondary antibodies (1:5000; Fude Biological Technology) for 1 h at room temperature (Song et al., 2012). The protein bands were revealed using an ECL system (Thermo Fisher Scientific), and the band densities were analyzed using ImageJ software.

### ELISA

Cell culture supernatants were collected, and IL-6, IL-10 and MCP-1 expression was analyzed using ELISA kits (R&D System), according to the manufacturer’s protocols.

### Immunofluorescence

Aortic valve cryosections (4.5 μm) were fixed with 4% paraformaldehyde for 15 min at room temperature. After blocking with 5% bovine serum albumin for 60 min, the slides were immunostained with primary antibodies against CD11c (1:150; Abcam, Cat. No. EP1347Y), CD206 (1:150; Abcam, Cat. No. ab8918), IL-37 (1:150; Abcam, Cat. No. ab153889). The slides were then stained with a Cy3- or FITC-labeled secondary antibody (Beyotime Biotechnology), and mounted with Vectashield antifade mounting media (Beyotime Biotechnology). Negative controls were performed by incubation with secondary antibody alone (omitting primary antibody). Nuclei were stained with Hoechst 33258 (Beyotime Biotechnology), cell membranes were stained with 3,3′-dioctadecyloxacarbocyanine perchlorate (DiO) (Beyotime Biotechnology) according to manufacturer’s instructions. Images were captured using fluorescence microscopy (Leica TCS-SP8 confocal microscope).

### Statistical Analysis

All data are presented as mean ±standard (SD). Statistical analysis was performed using SPSS 19.0 software. An unpaired, two-tailed Student’s *t*-test was used for two-group comparisons. A one-way analysis of variance (ANOVA) or two-way ANOVA with the SNK/Dunnett test was used to analyze the statistical significance among multiple groups. A *P*-value < 0.05 was considered statistically significant (^∗^*P* < 0.05, ^∗∗^*P* < 0.01, ^∗∗∗^*P* < 0.001).

## Results

### More M1 Macrophages Infiltrated in Calcified Aortic Valves, Accompanied by a Reduction in IL-37 Levels

**FIGURE 1:**
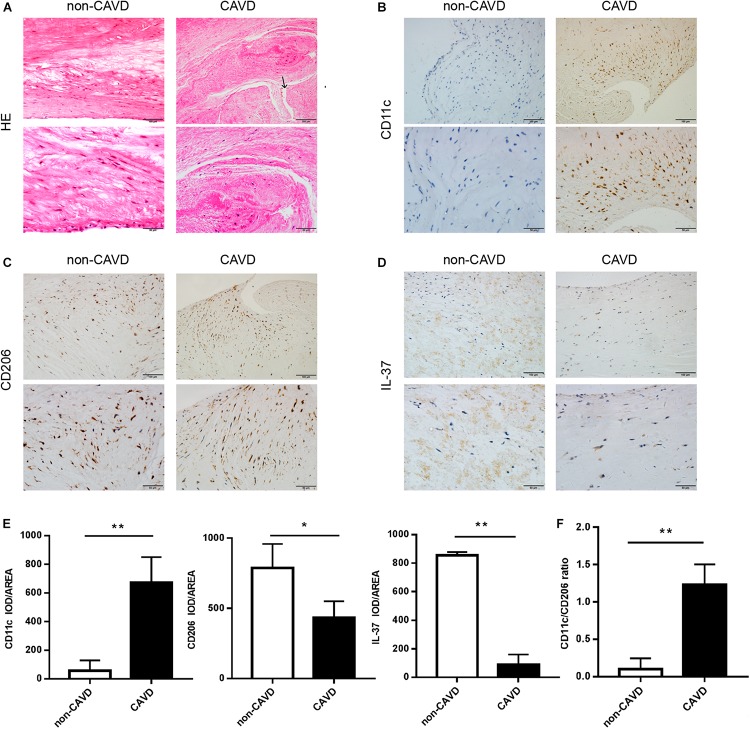
More M1 macrophages infiltrate in calcified aortic valves, with a reduction of IL-37 expression. **(A)** Normal and calcified human aortic valves were stained with HE. The arrow above shows the newly blood vessels in calcified aortic valves. Magnification, 200× and 400×; *n* = 6. **(B–D)** CD11c, CD206 and IL-37 expression are shown in calcified and non-calcified aortic valves. Magnification, 200× and 400×; *n* = 6. **(E)** Integrated optical density (IOD)/area of CD11c, CD206 and IL-37. Magnification, 400×, scale bar = 50 μm. **(F)** CD11c + cell-to-CD206 + cell ratio in CAVD and non-CAVD. Data are presented as the mean ±SD (*n* = 6); *P < 0.05, **P < 0.01. HE, hematoxylin-eosin; CAVD, calcific aortic valve disease; non-CAVD, non-calcific aortic valve disease.

Calcified aortic valves were collected from patients undergoing aortic valve replacement and normal aortic valves from patients undergoing heart transplantation surgery. Then the valves were stained with Hematoxylin and eosin (HE). The calcified valves showed marked fibrosis and neovascularization compared with the non-calcified valves (Figure 1A), which indicates a progression of tissue injury and fibrosis repair in CAVD. To detect M1 and M2 macrophage infiltration in the non-calcified and calcific valves, immunohistochemical staining was performed for CD11c (surface marker of M1) (Figure 1B) and CD206 (surface marker of M2) (Figure 1C). The infiltration of M1 macrophages (CD11c+) increased in calcified aortic valves (*P* < 0.01), while the accumulation of M2 macrophages (CD206+) of that decreased (*P* < 0.05) (Figure 1E). Moreover, calcified aortic valves showed more CD11c+/iNOS cells (M1 macrophages) infiltration than CD206+ cells (M2 macrophages), while non-calcified aortic valves showed the opposite (Supplementary Figures S1A, S2A). And the ratio of CD11c+/CD206+ cells evidently increased in calcified aortic valves compared with non-calcified aortic values (*P* < 0.01) (Figure 1F). In non-calcified aortic valves, IL-37 expressed both intracellularly and extracellularly (Supplementary Figure S1B). Valve tissues were also stained with the anti-IL-37 antibody (Figure 1D), showing that IL-37 expression decreased in calcified aortic valves (Figure 1E). Immunoblots of non-calcified and calcified aortic valve tissues also showed the same trend (Supplementary Figure S2B). These findings suggested that IL-37 may inhibit M1 but augment M2 infiltration. These results demonstrated that M1 macrophage levels increased in calcified human aortic valves compared with non-calcified aortic valves, which may be associated with a reduction in IL-37 levels.

### IL-37 Down-Regulated M1 Phenotype Markers and Pro-inflammatory Cytokine Levels

**FIGURE 2:**
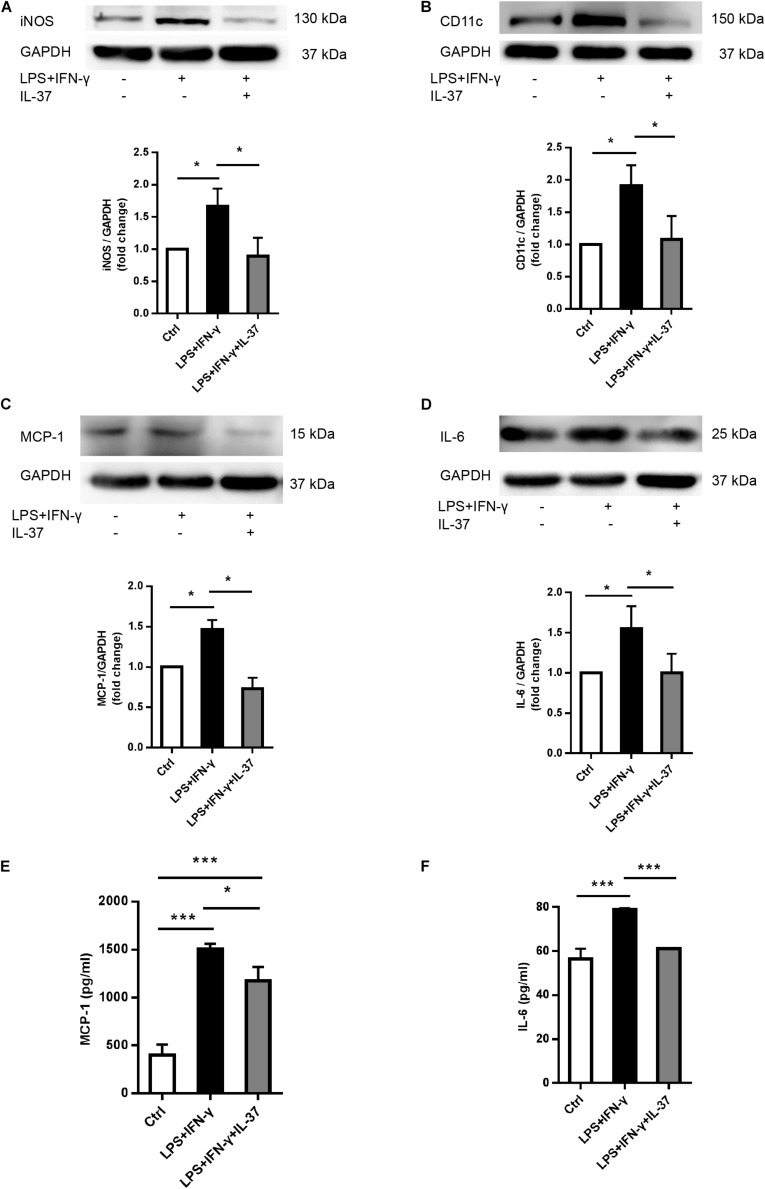
IL-37 suppresses M1 macrophage polarization and pro-inflammatory cytokines secretion. PMA-treated THP-1 cells were treated with LPS (100 ng/ml) and IFN-γ (20 ng/ml) in the presence or absence of recombinant IL-37 (0.1 ng/ml). **(A,B)** Representative immunoblots and densitometric data show that IL-37 inhibits iNOS and CD11c expression; *n* = 3, *P < 0.05. **(C,D)** Representative immunoblots and densitometric data show that IL-37 down-regulates MCP-1 and IL-6 expression; *n* = 3, *P < 0.05. **(E,F)** The ELISA data show that IL-37 down-regulates MCP-1 and IL-6 secretion; *n* = 3, *P < 0.05, ***P < 0.001.

To determine whether IL-37 suppresses M1 macrophage differentiation, we treated Thp-1 cells with PMA (100 ng/ml) to differentiate into resting (M0) macrophages, and then PMA-treated THP-1 cells were treated with LPS (100 ng/ml) and IFN-γ (20 ng/ml) in the presence or absence of recombinant IL-37. The levels of iNOS and CD11c increased significantly after treatment with LPS and IFN-γ. However, IL-37 markedly attenuated iNOS (Figure 2A) and CD11c (Figure 2B) expression in M1 macrophages. In addition, the levels of the pro-inflammatory cytokines MCP-1 and IL-6 decreased in the cell lysate (Figures 2C,D) and the supernatant (Figures 2E,F) after treatment with IL-37 in M1 macrophages. Taken together, these results demonstrate that IL-37 inhibits the differentiation of macrophages into the M1 phenotype and attenuates inflammation.

### IL-37 Up-Regulated M2 Phenotype Marker Expression

**FIGURE 3:**
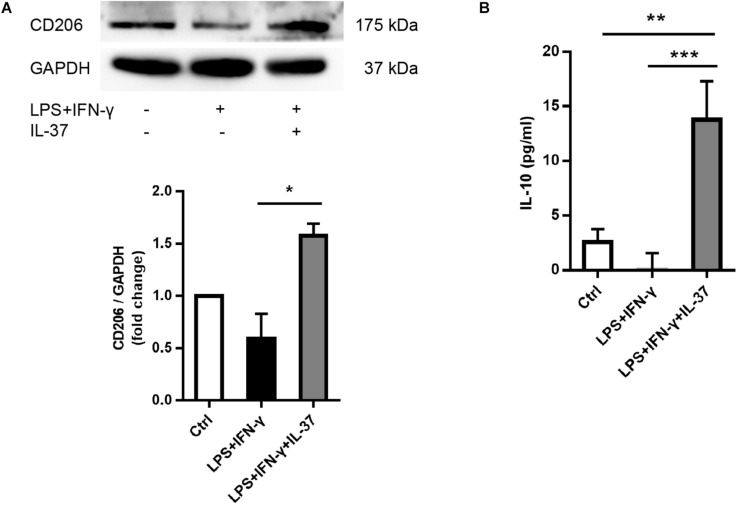
IL-37 promotes the expression of M2 macrophage markers.**(A)** Representative immunoblots and densitometric data show that CD206 expression increases after pre-treating PMA-treated THP-1 cells with recombinant human IL-37 (0.1 ng/ml) prior to stimulating with LPS (100 ng/ml) and IFN-γ (20 ng/ml); *n* = 3, *P < 0.05. **(B)** The ELISA data show that IL-37 up-regulates IL-10 secretion; *n* = 4, **P < 0.01, ***P < 0.001.

To determine the role of IL-37 in M2 polarization, we analyzed the cellular levels of the M2 macrophage markers CD206 and IL-10. PMA-treated THP-1 cells expressed less CD206 after exposed to LPS and IFN-γ. However, IL-37 increased CD206 expression (Figure 3A). ELISA showed that the levels of IL-10, an anti-inflammatory factor, were higher in the supernatant in the groups challenged by IL-37. However, the levels of IL-10 were very low in the group treated with LPS and IFN-γ without IL-37 (Figure 3B). These results indicated that IL-37 may somehow induce macrophages to differentiate into M2 macrophages.

### IL-37 Suppressed M1 Macrophage Polarization Through the NF-κB Pathway

**FIGURE 4:**
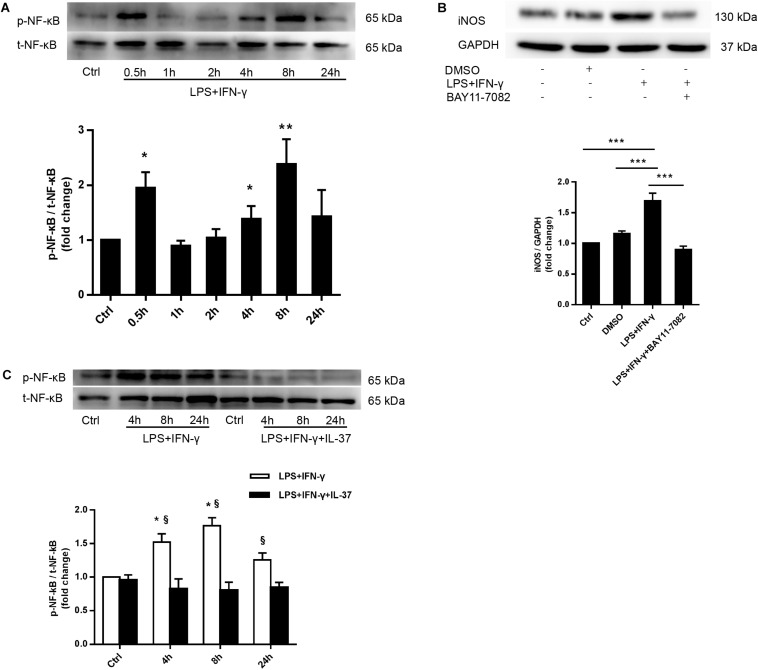
IL-37 suppresses M1 polarization through the NF-κB signaling pathway. **(A)** Representative immunoblots and densitometric data show that PMA-treated THP-1 cells exhibit enhanced NF-κB phosphorylation after treatment with LPS (100 ng/ml) and IFN-γ (20 ng/ml) for 0.5 to 24 h; *n* = 3, *P < 0.05, **P < 0.01 vs. corresponding control. **(B)** PMA-treated THP-1 cells were treated with BAY11-7082 (5 μM) 1 h prior to being treated with LPS (100 ng/ml) and IFN-γ (20 ng/ml). Representative immunoblots and densitometric data show that NF-κB pathway inhibition decreases iNOS expression in M1 macrophages; *n* = 3, ***P < 0.001. **(C)** PMA-treated THP-1 cells were treated with LPS (100 ng/ml) and IFN-γ (20 ng/ml) for 4–24 h in the presence or absence of recombinant IL-37 (0.1 ng/ml). Representative immunoblots and densitometric data show that treatment with recombinant IL-37 results in a reduction in NF-κB phosphorylation at different time point; *n* = 3, *P < 0.05 vs. corresponding control, §*P* < 0.05 vs. M1 macrophages treated with IL-37.

To elucidate the mechanism by which IL-37 suppresses M1 macrophage polarization, we detected the activation of NF-κB, an important signaling molecule that regulates macrophage polarization. We stimulated PMA-treated THP-1 cells with LPS and IFN-γ for 0-24 h and then examined NF-κB phosphorylation. As shown in Figure 4A, NF-κB p65 phosphorylation was markedly enhanced at 0.5 h. Interestingly, evidently enhanced NF-κB p65 phosphorylation was also detected at 4, 8, and 24 h but not at 1 h or 2 h. To further confirm the role of NF-κB in the modulation of M1 macrophage polarization, we pre-treated PMA-treated THP-1 cells with the NF-κB inhibitor BAY11-7082 an hour prior to treatment with LPS and IFN-γ for 24 h. As shown in Figure 4B, iNOS expression decreased after treatment with BAY11-7082. In the following study, we stimulated PMA-treated THP-1 cells with LPS and IFN-γ in the presence or absence of recombinant IL-37. We found that recombinant IL-37 reduced NF-κB phosphorylation at different time point except at 0.5 h (Figure 4C and Supplementary Figure S3). These observations imply that IL-37 could suppress M1 polarization through inhibiting the NF-κB pathway.

### IL-37 Modulated M1 Macrophage Polarization Through the Notch1 Signaling Pathway and Thus Modulated NF-κB Pathway Activation

**FIGURE 5:**
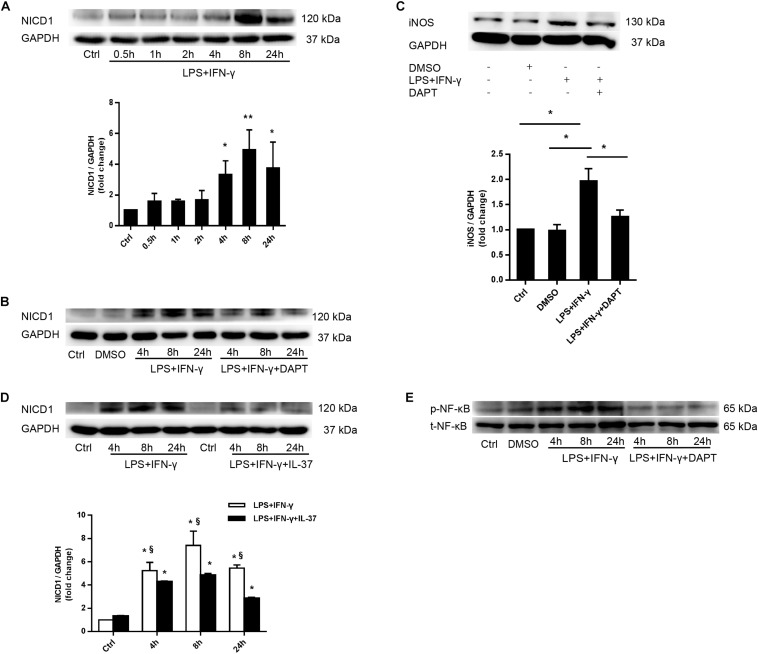
IL-37 modulates macrophage polarization through suppressing activation of the Notch1- NF-κB pathway axis. **(A)** Representative immunoblots and densitometric data show that stimulation with LPS and IFN-γ enhances Notch1 pathway activation in PMA-treated THP-1 cells; *n* = 3, *P < 0.05, **P < 0.01. **(B,C)** PMA-treated THP-1 cells were treated with DAPT (50 μM) 1 h prior to LPS (100 ng/ml) and IFN-γ (20 ng/ml) for 4–24 h. **(B)** Representative immunoblots show that DAPT inhibits Notch1 activation; *n* = 3. **(C)** Representative immunoblots and densitometric data show that Notch1 pathway inhibition reduces iNOS expression in M1 macrophages; *n* = 3, *P < 0.05. **(D)** PMA-treated THP-1 cells were treated with recombinant human IL-37 (0.1 ng/ml) 1 h prior to being treated with LPS (100 ng/ml) and IFN-γ (20 ng/ml) at different times. Representative immunoblots and densitometric data show that IL-37 decreases NICD1 expression at different time point in M1 macrophages; *n* = 3. *P < 0.05 vs. corresponding control, §*P* < 0.05 vs. M1 macrophages treated with IL-37. **(E)** Representative immunoblots show that Notch1 pathway inhibition decreases NF-κB phosphorylation; *n* = 3.

As shown in Figure 5A, LPS and IFN-γ co-treatment obviously induced the upregulation of Notch1 intracellular domain (NICD1) after 4 h. To determine whether the Notch1 signaling pathway modulates M1 macrophage polarization, we used DAPT, a γ-secretase inhibitor, to inhibit Notch1 intracellular domain (NICD1) generation (Figure 5B). Interestingly, treatment with DAPT evidently reduced iNOS levels in M1 macrophages (Figure 5C), indicating that the Notch1 pathway plays an important role in mediating M1 macrophage polarization.

To further explore whether IL-37 suppresses M1 macrophage polarization through the Notch1 pathway, we treated PMA-treated THP-1 cells with recombinant IL-37 (0.1 ng/ml) 1 h prior co-treatment with LPS and IFN-γ for 4–24 h. The immunoblot showed that IL-37 decreased Notch1 pathway activation at different time points and markedly reduced NICD1 production (Figure 5D). These findings indicate that IL-37 could modulate M1 macrophage polarization through Notch1 pathway.

Our previous study have shown that Notch1 modulates the inflammatory response in the interstitial cells of human aortic valves through LPS-induced NF-κB pathway (Zeng et al., 2012). To determine the role of Notch1 in NF-κB activation, we treated PMA-treated THP-1 cells with DAPT before co-treatment with LPS and IFN-γ. NF-κB phosphorylation was significantly reduced by Notch1 pathway inhibition (Figure 5E). In addition, as shown in Figures 4A, 5A, after treatment with LPS and IFN-γ, NF-κB activation was consistent with Notch1 activation from 4–24 h. These evidences indicated that the Notch1 signaling pathway modulates NF-κB pathway activation to regulate M1 macrophage polarization. Therefore, IL-37 could decrease NF-κB activation through suppressing the Notch1 pathway.

## Discussion

Chronic inflammation and osteogenic activity in the aortic valve leaflet tissue contribute to CAVD (Rajamannan et al., 2011; Natorska and Undas, 2015). Infiltration of macrophages, particularly M1 macrophages, promotes CAVD mainly through the cross-talk with aortic valve interstitial cells (AVICs) (Lee and Choi, 2016). The present study uncovered an anti-inflammation function of IL-37 through suppression of M1 macrophages. IL-37, expressed in human but not in murine, has been proved possessing an anti-inflammatory potential in innate and adaptive immunity both *in vitro* and *in vivo* (Nold et al., 2010).

In the present study, we demonstrated that calcific aortic valves showed more M1 but less M2 macrophage infiltration with deficiency in IL-37, compared with normal aortic valves. Notably, recombinant human IL-37 suppressed M1 polarization and expression of pro-inflammatory cytokines. Further, differentiating M1 macrophages into M2 macrophages may be induced by the micro environment created by IL-37. Mechanistic evidences showed that IL-37 attenuated M1 polarization through the inhibition of Notch1 and NF-κB activation, the pathways that regulate the polarization of macrophages (Wang Y. C. et al., 2010; Adamson et al., 2016). It should be noted that the Notch1- NF-κB axis activation was involved in M1 polarization, which could be suppressed by IL-37.

Here, we showed that calcified human aortic valves expressed lower levels of IL-37 protein and that this alteration in cellular IL-37 levels might be associated with the more infiltration of M1 macrophages but less of M2 in diseased aortic valves. Staining of Hematoxylin and eosin showed that the formation of new blood vessels promoted and the elastic fibers were in degeneration and disorder in diseased valves. Further, the ratio of M1 to M2 macrophages markedly increased in calcified aortic valves, with a lower expression of IL-37, indicating that levels of IL-37 in aortic valves may play a role in macrophage polarization. *In vitro* experiments, treatment with IL-37 effectively suppressed M1 macrophage polarization and thus reduced the expression of pro-inflammatory cytokines, such as IL-6 and MCP-1. However, M2 macrophages and its secreting anti-inflammatory cytokine IL-10 increased when exposed to recombinant IL-37. These lines of evidences suggest that IL-37 plays an important role in suppression of M1 macrophage polarization and may promote the differentiation of M2 macrophages.

IL-37 suppresses M1 macrophage polarization and thus exerts anti-inflammatory effect, which may attenuate the calcified process of CAVD. CAVD is a slowly progressing disorder characterized by lipoprotein deposition, chronic inflammation, and active leaflet calcification (Freeman and Otto, 2005). In normal valves, aortic valvular endothelial cells (AVECs) and aortic valvular interstitial cells (AVICS) are important to maintain the homeostasis in the aortic valves (Combs and Yutzey, 2009). Impairment of valvular endothelium due to damage to AVECs can result in accumulation of lipoproteins (LDL) in the subendothelial space. Oxidized LDL (oxLDL), derived from LDL molecules, can trigger macrophage infiltration into aortic valves by increasing cell adhesion molecules, such as ICAM-1 and VCAM-1 (Mohty et al., 2008). These infiltrating macrophages release pro-inflammatory cytokines, including IL-6 and TNF-α, which differentiate AVECs into AVICs via endothelial-mesenchymal transition (Mahler et al., 2013; Lee and Choi, 2016). AVICs, the most abundant cell type in aortic valves, are capable of differentiating into myofibroblasts and osteoblast-like cells in specific conditions, which promote fibrosis and valve calcification, respectively (Rajamannan et al., 2011; Lee and Choi, 2016). Osteoblast-like AVICs promote aortic calcification through a mechanism similar to osteogenesis (Rajamannan et al., 2003). In calcified aortic valves, more monocytes infiltrate into the tissue to differentiate into pro-inflammatory macrophages, which secret inflammatory cytokines such as IL-6 and TNF-α, promoting AVICs activation and inducing alkaline phosphatase expression, eventually leading to aortic valvular calcification (Kaden et al., 2005; Lee and Choi, 2016). Thus, through reducing the inflammatory cytokines produced by M1 macrophages, IL-37 may inhibit the osteogenic activity of AVICs and therefore alleviate the calcification.

Here, we demonstrated that IL-37 suppresses M1 polarization and may convert M1 into M2 macrophages. Recombinant human IL-37 suppressed the switch response to the TLR4 agonist (LPS) and IFN-γ, which inhibits PMA-treated THP-1 cells to polarize into M1 macrophages. In CAVD, M1 macrophages play a pro-inflammatory role, with the expression of inflammatory cytokines including MCP-1, IL-6 and TNF-α whereas M2 macrophages play an anti-inflammatory role, secreting anti-inflammatory cytokine IL-10 (Lawrence and Natoli, 2011; Mantovani et al., 2013; Liu et al., 2014). It has been demonstrated that macrophages exhibit functional plasticity and M1 and M2 macrophages transform into each other as a result of changes in the microenvironment in infectious diseases, atherosclerosis and calcific aortic diseases (Gordon and Martinez, 2010; Liu et al., 2014). Notably, a recent research reports that IL-37 directly suppresses M1 polarization in Concanavalin A induced liver injury C57BL/6 mice while promotes M2 polarization indirectly by increasing the expression of IL-4 and IL-13 (Feng et al., 2019). In this study, we showed that IL-37 attenuated the inflammation induced by M1 macrophages and may convert M1 into M2 macrophages, and thus had a potential to dampen the calcification of CAVD.

Several studies have demonstrated that the Notch1 and NF-κB pathways induce macrophages to differentiate into M1 (Palaga et al., 2008; Wang Y. et al., 2010; Zhang et al., 2015; An et al., 2017). To investigate the effects of TLR on Notch receptor expression, the researchers treats the macrophage-like cell line RAW264.7 with LPS, IFN-γ or LPS plus IFN-γ. These treatments result in the significant up-regulation of Notch1 transcription. Treatment by LPS plus IFN-γ is the strongest inducer of the up-regulation of Notch1 transcription (Palaga et al., 2008). The forced activation of Notch signaling augments M1 polarization regardless of whether M1 or M2 inducers are applied. Inhibition of Notch signaling causes M2 polarization even in the presence of M1 inducers (Wang Y. et al., 2010). It seems that the Notch signaling pathway is essential for M1 polarization.

In this study, we noted that recombinant human IL-37 suppressed M1 polarization by inhibiting NF-κB phosphorylation and Notch1 activation. Interestingly, suppression of Notch1 activation evidently decreased NF-κB pathway phosphorylation, indicating that the Notch1 pathway modulated NF-κB activation. Our previous study showed that recombinant human IL-37 suppresses NF-κB phosphorylation and inhibits ERK1/2 phosphorylation in AVICs exposed to LPS, Pam3CSK4, or oxLDL (Zeng et al., 2017). Notch1 modulates NF-κB activation to mediate complex responses in both macrophages and AVICs (Palaga et al., 2008; Zeng et al., 2013). Thus, inhibiting Notch1 with IL-37 alleviates Notch1 and NF-κB activation to efficiently suppress M1 polarization and attenuate inflammation.

**FIGURE 6:**
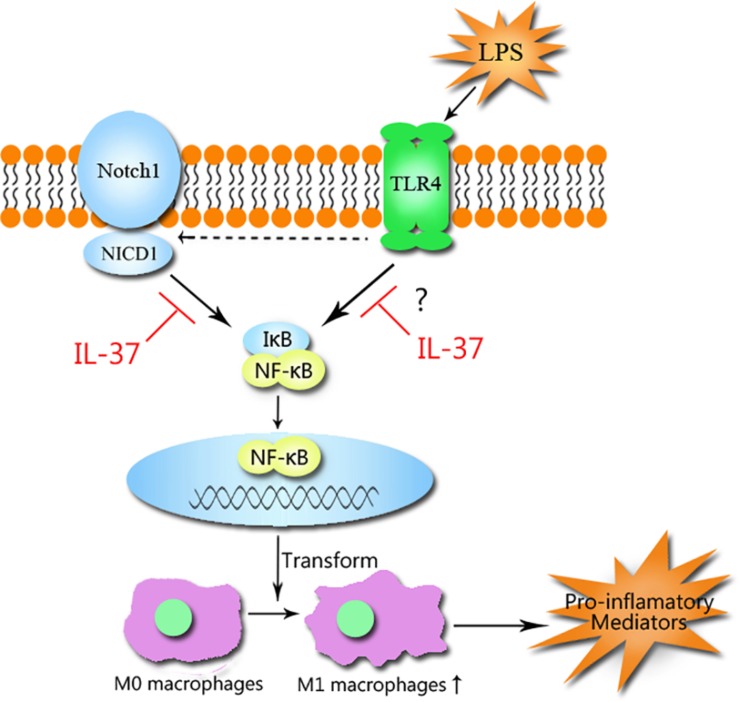
The mechanism that IL-37 suppresses M1 macrophage polarization. IL-37 suppresses M1 macrophage polarization through the Notch1-NF-κB axis pathways. The activation of Notch1 by TLR4 stimulation with LPS causes the generation of NICD1, which interacts with IκB and results in the activation of NF-κB, but the generation can be inhibited by IL-37. IL-37 prevents Notch1 and NF-κB activation and thus attenuates M1 polarization and inhibits the inflammatory response.

Our previous study showed that stimulating TLR4 with LPS induces the release of Jagged1, a Notch1 ligand. Activation of Notch1 by Jagged1 causes the generation of NICD1, which enhances NF-κB activation by interacting with IKK, resulting in the augmentation of the inflammatory response (Zeng et al., 2012). Activation of Notch signaling augments M1 polarization, while inhibition of the signaling way increases M2 polarization (Wang Y. et al., 2010). Based on these findings and the evidence collected by this study, we demonstrate that IL-37 suppresses M1 polarization mainly by inhibiting the Notch1- NF-κB axis activation (Figure 6). However, whether IL-37 attenuates M1 polarization directly by inhibiting NF-κB activation deserves further investigation.

Notably, IL-37 exerts anti-inflammatory function through both extracellular and intracellular pathways. To exert anti-inflammatory effects, extracellular IL-37 binds to IL-18Rα and then the co-receptor IL-1R8 (formerly SIGIRR) is recruited to form the tripartite ligand-receptor complex IL-37-IL-1R8-IL-18Rα to suppress NF-κB function (Lunding et al., 2015). Also, the complex augments the inhibition of the production of IFN-γ and thus suppresses M1 polarization (Nold et al., 2010; Nold-Petry et al., 2015). IL-37 triggers inhibition of several signaling pathways, such as the Notch1, NF-κB, mTOR, and MAPK pathways, thereby alleviating M1 polarization. However, the protein induces an increase in the activity of anti-inflammatory pathways, such as the STAT6, Mer and AMPK pathways, resulting in M2 polarization (O’Neill and Hardie, 2013; Nold-Petry et al., 2015). Meanwhile, IL-37 translocates to the nucleus after caspase-1 processing and interacts with the transcription factor Smad3 in LPS-stimulated macrophages to exert its anti-inflammatory effects (Nold et al., 2010; Garlanda et al., 2013). Thus, we assume that IL-37 exerts an influence on these signaling pathways in macrophages to modulate polarization and attenuate inflammation.

The present study has limitations. In this study, we focused on the regulation of IL-37 on M1 polarization of macrophages. However, whether IL-37 could directly induce M2 macrophage polarization or switch M1 phenotype into M2 and its mechanism is still unclear. For further investigation, we will make a deeper research on the regulation of IL-37 in M2 polarization.

## Conclusion

In summary, our study showed that calcific aortic valve leaflets exhibit a reduction in IL-37 and more M1 infiltration than normal aortic valves and thus exist in a pro-inflammatory state. The study also showed that recombinant IL-37 suppresses M1 macrophage polarization through Notch1-NF-κB axis pathway, thereby exerting a marked anti-inflammatory effect. Totally, these findings demonstrate that IL-37 has the potential to attenuate CAVD progression by suppressing M1 macrophage polarization.

## Data Availability Statement

All datasets generated for this study are included in the article/Supplementary Material.

## Ethics Statement

The studies involving human participants were reviewed and approved by the Ethical Committee of Nanfang Hospital, China. The patients/participants provided their written informed consent to participate in this study.

## Author Contributions

PZ, SS, XY, SZ, XM, DZ, DX, and QZ conceived and designed the experiments. PZ, QL, WD, SZ, and QM performed the experiments. PZ, QL, and SZ analyzed the data. PZ, WD, SZ, DX, and QZ wrote the manuscript.

## Conflict of Interest

The authors declare that the research was conducted in the absence of any commercial or financial relationships that could be construed as a potential conflict of interest.
