# Supplementary material for: Interleukin 37 Suppresses M1 Macrophage Polarization Through Inhibition of the Notch1 and Nuclear Factor Kappa B Pathways
Source: Front Cell Dev Biol. 2020 Feb 14;8:56. doi: 10.3389/fcell.2020.00056 (PMC7033589; doi:10.3389/fcell.2020.00056)
Supplement: Supplementary file 1 [file Table_1.docx]

Supplementary Material

## Supplementary Figures


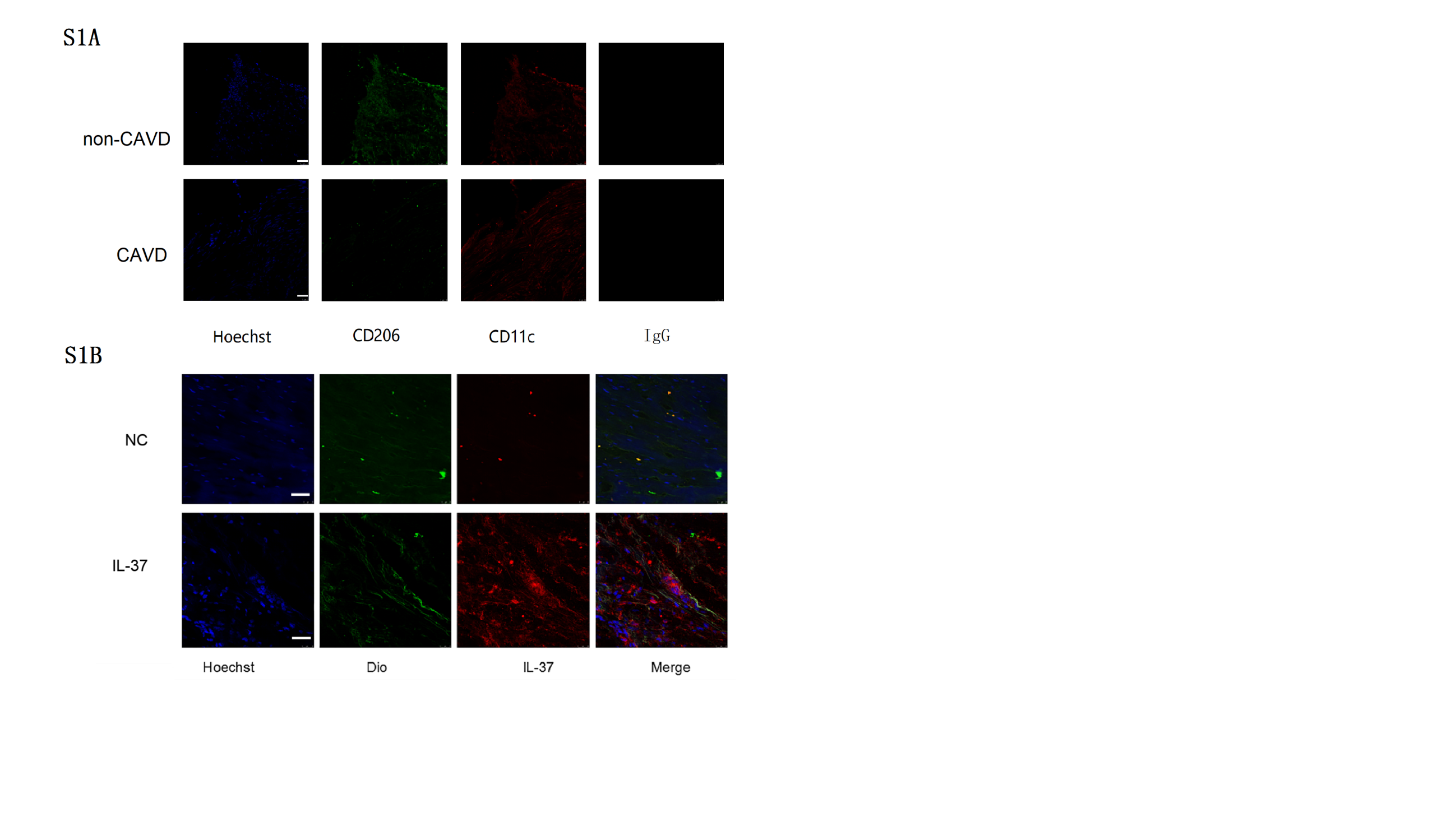


##

**Supplementary Figure 1.** Calcified aortic valves show more M1 infiltration than M2 while non-calcified aortic valves show the opposite. **(A)** Representative images show that there are less CD11c+ cells (M1 macrophages) than CD206+ cells (M2 macrophages) accumulation in non-calcified aortic valves, while calcified aortic valves show more CD11c+ cells (M1 macrophages) than CD206+ cells . **(B)** IL-37 staining in non-calcified aortic valves indicates that IL-37 locates both intracellularly and extracellularly. IF, Immunofluorescence; IgG: isotype control,NC, negative control; Dio, 3,3′-dioctadecyloxacarbocyanine perchlorate; scale bar, 25μm.

**
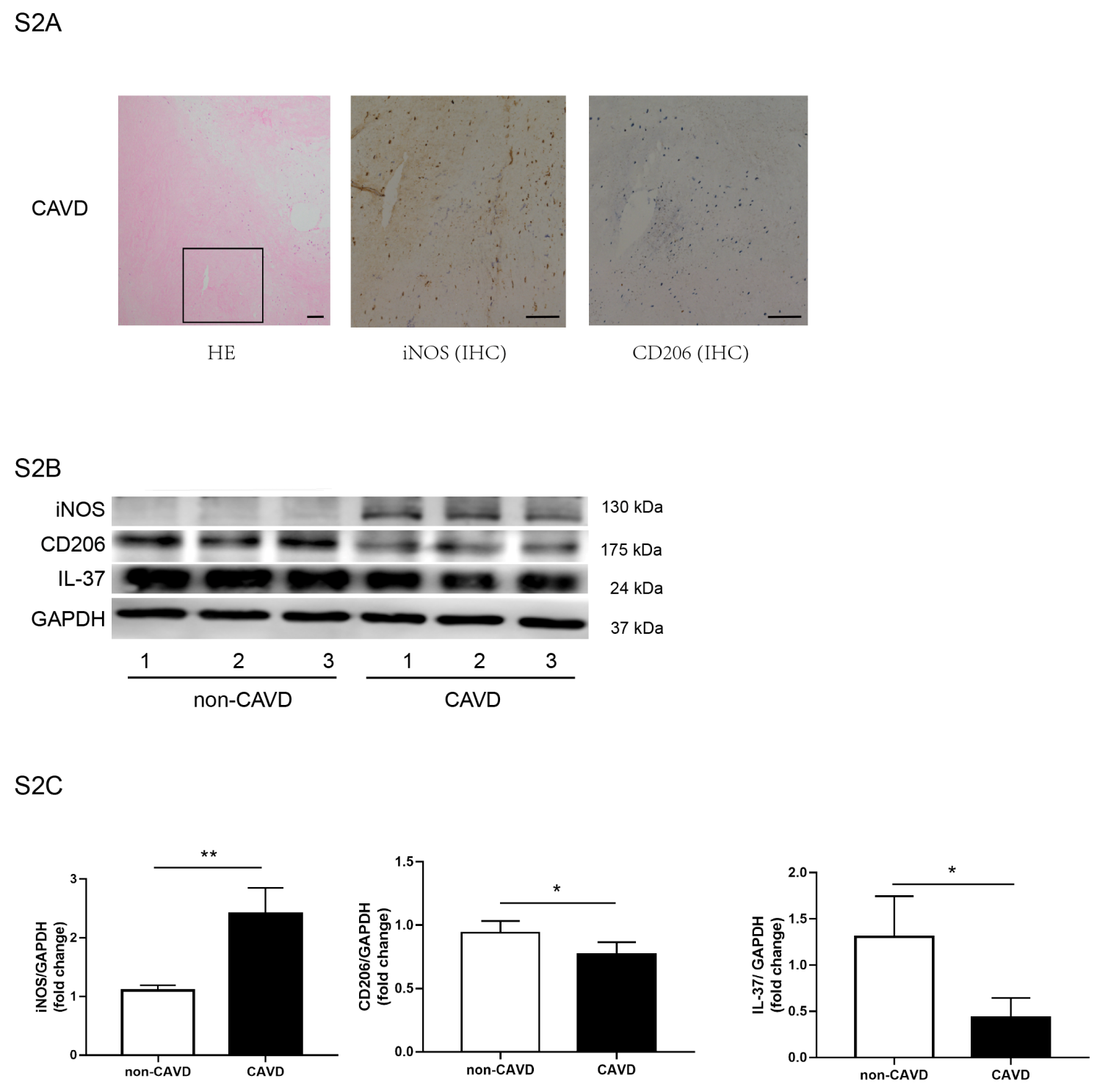
**

**Supplementary Figure 2.** Calcified aortic valve tissues show more M1 macrophage infiltration and less IL-37 expression. **(A)** Representative images show that there are more M1 macrophage infiltration and less CD206 expression in calcified aortic valve; scale bar, 100μm. HE, hematoxylin-eosin; IHC, Immunohistochemistry. **(B)** Representative western blots show the expression of iNOS (M1 marker), CD206 (M2 marker) and IL-37 in non-calcified and calcified aortic valves; n=3. **(C)** Densitometric data show the expression of iNOS , CD206 , IL-37 in non-calcified and calcified aortic valve tissues; *P < 0.05, **P < 0.01.


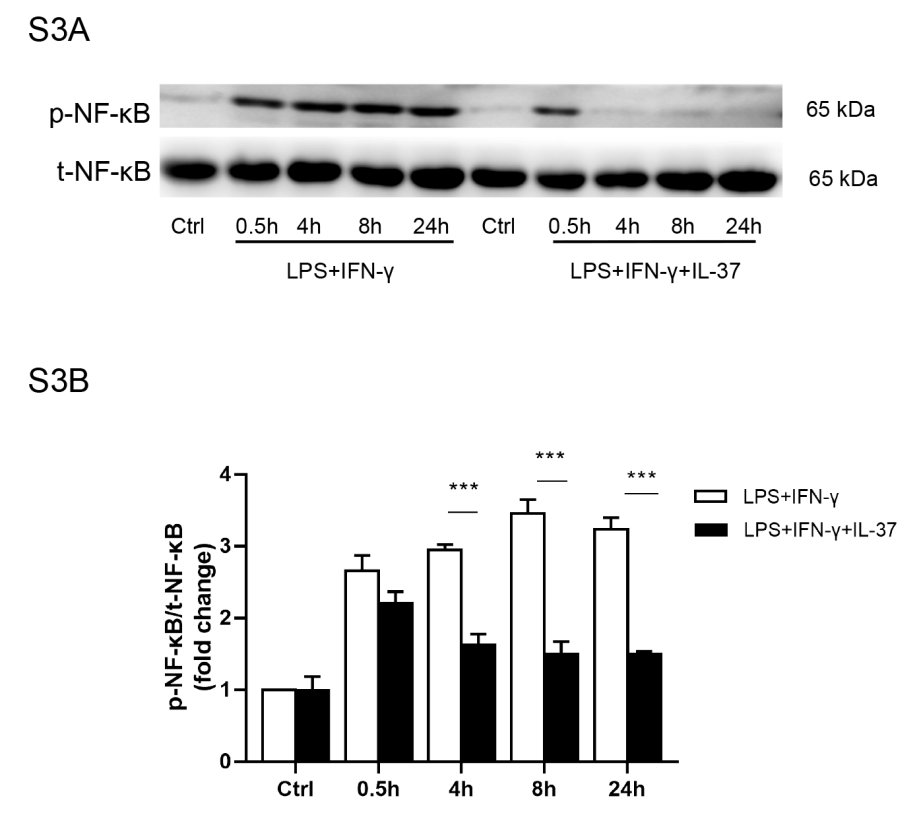


**Supplementary Figure 3.** Recombinant human IL-37 inhibits NF-κB activation induced by LPS and IFN-γ co-treatment. **(A-B)** Representative western blots and densitometric data show that IL-37 inhibits NF-κB phosphorylation at different time points; n=3; ***P < 0.001.
